# Anti-oxidative stress therapies prevent severe chemotherapy-induced peripheral neuropathy in colorectal cancer patients treated with oxaliplatin: a systematic review and meta-analysis

**DOI:** 10.3389/fonc.2025.1642552

**Published:** 2025-09-10

**Authors:** Mohamed Salama, David Barnes, Anni Georghiou, Mariam Murad, Seham Almalki, Zubair Ahmed, Mark R. Openshaw, Claire Palles, Richard I. Tuxworth

**Affiliations:** ^1^ Birmingham Centre for Neurogenetics, University of Birmingham, Birmingham, United Kingdom; ^2^ Department of Cancer and Genomic Sciences, School of Medical Sciences, College of Medicine and Health, University of Birmingham, Birmingham, United Kingdom; ^3^ University Hospitals Birmingham NHS Foundation Trust, Edgbaston, Birmingham, United Kingdom; ^4^ Department of Inflammation and Ageing, School of Infection, Immunity and Inflammation, College of Medicine and Health, University of Birmingham, Birmingham, United Kingdom; ^5^ Department of Biotechnology, Faculty of Science, Taif University, Taif, Saudi Arabia; ^6^ Birmingham NIHR Biomedical Research Centre, University of Birmingham, Birmingham, United Kingdom

**Keywords:** bowel cancer, CIPN, platinum agents, neurotoxicity, oxidative stress

## Abstract

**Background and purpose:**

Chemotherapy-induced peripheral neuropathy (CIPN) is a major side-effect of many commonly used cancer drugs, affecting up to 90% of patients treated with oxaliplatin. This systematic review and meta-analysis analysed randomised controlled trials (RCTs) to determine if any pharmacological agents or traditional medicines can prevent oxaliplatin-induced peripheral neuropathy (OIPN) in colorectal cancer (CRC) patients.

**Materials and methods:**

We searched PubMed, EMBASE and Web of Science for RCTs published before March 2025 that included patients with CRC who received oxaliplatin-based chemotherapy and had peripheral neuropathy quantified using Common Toxicity Criteria for Adverse Events (CTCAE). Meta-analysis was performed for agents tested in three or more RCTs with a minimum combined sample size of 100 patients.

**Results:**

20 studies were included in the systematic review with a median sample size of 61 (range 14-2450). Meta-analysis was conducted for two treatments: first, agents with anti-oxidative stress properties and second, Ca^2+^/Mg^2+^ infusions. Anti-oxidative stress treatments were associated with a significant reduction of grade ≥2 OIPN at the end of treatment (OR:0.04, 95%CI:0.01-0.12; p<0.00001). No reduction of grade ≥2 OIPN was observed for Ca^2+^/Mg^2+^ infusions. 35% of studies had potential high risk of bias and 45% of studies showed low risk of bias.

**Conclusions:**

Whilst the existing published RCTs included small numbers of patients, the meta-analysis indicates that anti-oxidative stress therapies can prevent severe OIPN developing at the end of treatment in CRC patients. A large, randomised, placebo-controlled trial assessing OIPN using CTCAE grades and patient-reported outcomes is warranted to confirm these findings.

## Introduction

Chemotherapy-induced peripheral neuropathy (CIPN) is one of the most frequent side-effects of commonly used anti-neoplastic agents, including platinum drugs, taxanes and proteosome inhibitors. 30-50% of patients experience CIPN with low dose regimens but at higher doses as many as 90% of patients experience neuropathy making it an important dose limiting toxicity (1). Symptoms include pain in the fingers and toes, loss of sensation, cold or mechanical allodynia and mechanical weakness. Symptoms can be life long, and in severe cases life changing. Motor symptoms are common, and many patients also report long-lasting cognitive effects. Neurotoxicity is severe in many cases, forcing tapering of chemotherapy regimens or even cessation of treatment, thereby limiting the efficacy of cancer treatment (2).

Oxaliplatin, a third-generation platinum compound, is one of the most commonly used chemotherapeutic agents for treating colorectal cancer (CRC) (3). It can cause acute neuropathy during, or immediately after infusion and symptoms of oxaliplatin-induced peripheral neuropathy (OIPN) can also emerge weeks or months later after the completion of chemotherapy (4). The severity of symptoms is usually proportional to the cumulative dose of the drug and therefore progressively worsens during therapy (5). A recent meta-analysis in patients treated for CRC found that 58% of patients reported OIPN 6 months after treatment, 45% at 12 months, 32% at 24 months, and 24% at 36 months (6). Patients treated with oxaliplatin often exhibit a coasting phenomenon in which OIPN symptoms continue to worsen for several months after treatment (7). Platinum drugs generate DNA damage in the nucleus but mitochondria are also affected and several studies have suggested mitochondrial damage is central to OIPN (8).

CRC is now the third most common cancer in the UK and the second most common in males aged 45 to 74 years and females aged 45 to 54 years (9). Oxaliplatin-based treatment is the standard adjuvant treatment for stage III CRC and is a standard first line treatment for metastatic CRC patients. Hence, there is an urgent need to find therapies that can prevent severity OIPN from occurring. No current therapies are effective at preventing OIPN; duloxetine, venlafaxine, gabapentin, pregabalin, lamotrigine, and amitriptyline are commonly given as initial treatments for neuropathic pain, but these drugs are only of limited benefit (10).

Three systematic reviews have previously been published examining the evidence for the utility of pharmacological interventions in preventing incidence of peripheral neuropathy induced by chemotherapy agents. Two included studies treating cancer patients with any chemotherapeutic agent (11, 12) and one focused specifically on studies treating with oxaliplatin (13). Hershman et al., 2014 (11) reviewed studies published before April 2013, and Loprinzi et al., 2020 (12) reviewed studies published between 2013 and 2020. Both concluded that there are no pharmacological interventions that can currently be recommended to prevent CIPN. Hershman et al. concluded that there was strong evidence to recommend against the use of acetyl-L-carnitine, diethyldithiocarbamate, nimodipine and moderate evidence against using amifostine, amitriptyline, Ca^2+^ and Mg^2+^ infusions, Org 2766, all-trans retinoic acid, recombinant human leukaemia inhibitory factor (rhuLIF) and vitamin E, or glutathione in paclitaxel/carboplatin-treated patients only. Loprinzi et al. found a lack of evidence of benefit for use of calmangafodipir, cannabinoids, carbamazepine, L-carnosine, gabapentin/pregabalin, glutamate, goshajinkigan, metformin, minocycline, N-acetylcysteine, omega-3 fatty acids, oxcarbazepine, rhuLIF, venlafaxine, and vitamins B and E.

Peng et al., 2022 (13) restricted their review to trials of oxaliplatin therapy published before August 2020 and trials of multiple cancer types were included. The evidence for 29 pharmacological interventions aiming to reduce OIPN was reviewed and 2 interventions were subsequently analysed by meta-analyses of 2 and 3 studies, respectively: N-acetylcysteine and glutathione. Both treatments were associated with a lower risk of common toxicity criteria (CTCAE) grade ≥2 OIPN. However, of the 5 studies included in the meta-analyses, 4 were assessed as having unclear or high risk of bias, making interpretation difficult. Peng et al. (13) also noted substantial differences in timing and/or scoring methods used to assess the severity of OIPN between trials, and that many trials were not double-blind, randomised, placebo-controlled, all of which make analysis difficult or impossible.

Here, we report the results of a new systematic review of pharmacological interventions assessed for their ability their ability to prevent OIPN in a clinical trial including CRC patients and published before March 2025. Among other strict inclusion criteria, we only analysed studies that had assessed OIPN using the CTCAE scale and where possible, we performed sensitivity analyses excluding trials where it was unclear if blinding had been applied. Meta-analysis was only performed for interventions investigated by three or more independent studies.

## Materials and methods

### Search strategy

The PubMed, EMBASE and Web of Science databases were searched in March 2025 for peer-reviewed English-language randomised controlled trials (RCTs). Searches were not limited by date restrictions. Search terms were: “Oxaliplatin induced peripheral neuropathy treatment” OR “Oxaliplatin induced neurotoxicity treatment” OR “Chemotherapy induced peripheral neuropathy and therapy” OR “Chemotherapy induced neurotoxicity and therapy”. Additional references were also obtained from the references cited by a recent relevant meta-analysis (13). Two investigators (M.S. and Z.A.) independently read and selected the retrieved abstracts. Discrepancies between the reviewers’ selections were resolved by discussion. Full text versions of potentially eligible studies were then read by M.S. to confirm each conformed to the inclusion and exclusion criteria.

### Inclusion criteria

RCTs were included if the worst OIPN grade on treatment in adult CRC patients was evaluated using the CTCAE scale and patients were randomised to a pharmacological agent or traditional herbal medicine being tested as a preventative treatment for OPIN. To be included in the meta-analysis, studies needed to report how many participants in each arm had experienced either <grade 2 OIPN or ≥grade 2 OIPN and specify the time point at which this was measured. The CTCAE version used in each study was not considered since the scoring criteria are consistent across all versions.

### Exclusion criteria

Studies were excluded if: there was no full text published in English; OIPN was only evaluated using an alternative to the CTCAE scale; it was not possible to calculate counts <grade 2 and ≥grade 2 OIPN (n=4) (14–16); analysis included patients with baseline peripheral neuropathy (PN) (n=2) (17, 18) the study aimed to treat rather than prevent PN (n=1) (19) patients with ECOG performance status 3 were included in the analysis (n=1) (20); or ≤10 patients were available for analysis (n=1) (21).

### Timepoints when OIPN was assessed

Different trials assessed OIPN at different time points. Where available, we extracted data from trials that assessed OIPN grade midway through treatment (cycles 4-6) and at the end of treatment (cycles 8-12) separately. In the Ca^2+^/Mg^2+^ infusion trials (22–25), highest PN CTCAE grades on treatment were provided. We combined results for CTCAE grade 2 and above since grade 2 is the usual threshold for clinical interventions, such as dose reduction of chemotherapy or treatment deferral.

### Data extraction and quality assessment

Data was extracted from the 20 included studies by two of 5 investigators independently (M.S., M.M, D.B., A.G. and C.P.). Data was imported into RevMan (26).

### Meta-analysis

Inverse weighted random effects meta-analysis was performed in RevMan (26) for pharmacological agents or classes of drugs if there were data from a minimum of 100 patients across 3 or more studies. Heterogeneity was estimated using the restricted maximum likelihood method within RevMan. Risk of bias was also performed using RevMan, assisted by the RoB-2 tool (27).

## Results

### Study selection and included interventions

**Figure 1:**
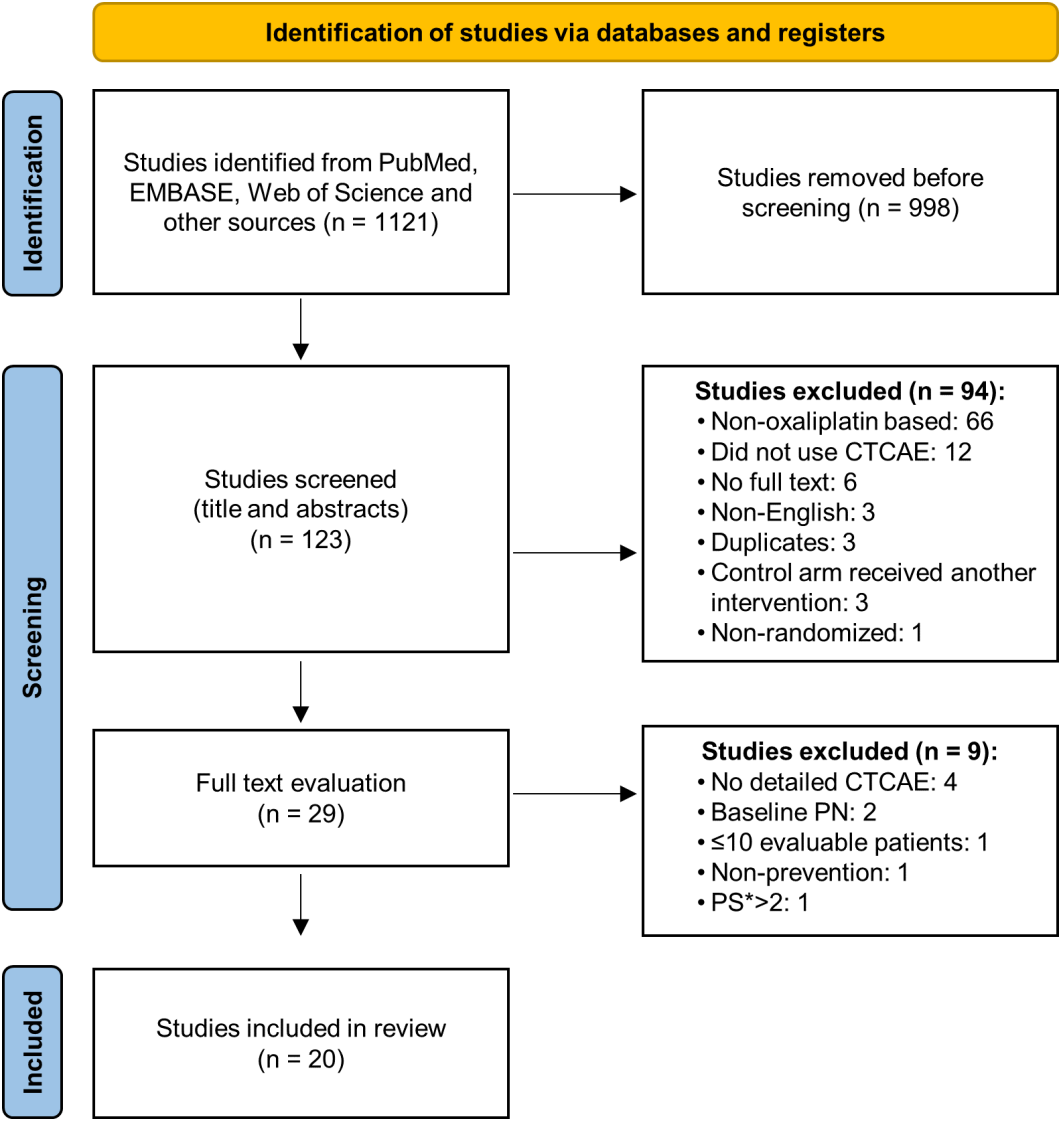
PRISMA flow diagram showing how studies were reviewed and selected. Identification, screening and exclusion criteria provided. The numbers of publications excluded at each step, and the reasons for this are indicated. CTCAE, Common Toxicity Criteria Adverse Event; PN, peripheral neuropathy; PS, Performance status.

**Figure 1** shows the PRISMA flow chart of how RCTs were identified and reviewed. We reviewed the full-text report of 29 studies (**Supplementary Table 1**), of which 20 met the inclusion criteria (summarised in **Table 1** with full details supplied in **Supplementary Table 2**). 12 studies were double-blinded and placebo controlled (22–25, 28–35), three were placebo controlled but blinding was unclear (37–39) and five studies compared to a control arm with no or unclear blinding (36, 40–43). Median incidence of grade ≥2 OIPN, as measured at the end of treatment (8-12 cycles), was 57.95% (range 31.2-100%) in the placebo or control arms.

**Table 1:** Summary of included trials.

Author	Study population	Regimen dose of oxaliplatin treatment schedule treatment intent	Pharmacological Intervention tested (N)	Comparison arm (N)	Timepoints when ≥grade 2 OIPN was assessed
Bondad et al, 2020 (28)^Δ^	•Country: Iran. • Ethnicity: not provided. • Age (median and range): overall: 59.5 (52–65.5), intervention: 57 (52–63), control: 62.5 (53–67). • Sex: M: F: 17/15; intervention: 7/9; control: 10/6. • Cancer types: CRC (N=24), gastric (N=8); intervention: 11 CRC and 5 gastric; control: 13 CRC and 13 gastric, • Stage of disease: not provided. • PS: not provided.	XELOX 130 mg/m^2^ Every 3 weeks Adjuvant	N-Acetylcysteine* (16)	Placebo (16)	Baseline and at each 3-week cycle
Cascinu et al, 2002 (29)	• Country: Italy. • Ethnicity: not provided. • Median age: intervention: 65, control: 65. • Sex: M: F: 31/21; intervention: 12/14; control: 19/7. • Cancer types: CRC. • Stages of disease: IV. • (PS: 0): intervention (N=17); control (N=20), (PS: 1): intervention (N=9); control (N=9).	FOLFOX 100 mg/m^2^ Every 2 weeks Palliative	Reduced glutathione* (26)	Placebo (26)	Baseline, after 4, 8 and 12 cycles
El-Fatatry et al, 2018 (36)	• Country: Egypt. • Ethnicity: not provided. • Median age: not provided. • Sex: not provided. • Cancer types: CRC. • Stages of disease: III. • PS: 0-1.	FOLFOX4 85 mg/m^2^ Every 2 weeks Adjuvant	Metformin* (20)	Control (20)	Baseline and at each 2-week cycle
Lin et al, 2006 (37)	• Country: China. • Ethnicity: not provided. • Age (median and range): overall: 63.5 (41–78), intervention: 58 (41-75), control: 65 (43-78). • Sex: M: F: 9/5; intervention: 4/1; control: 5/4. • Cancer types: CRC. • Stages of disease: III. • (PS: 0): intervention (N=4); control (N=8), (PS: 1): intervention (N=1); control (N=1).	FOLFOX4 85 mg/m^2^ Every 2 weeks Adjuvant	N-Acetylcysteine* (5)	Placebo (9)	Baseline and at each 2-week cycle
Milla et al, 2009 (38)	• Country: Italy. • Ethnicity: not provided. • Age (median and range): overall: 61 (44-75). • Sex: M: F: 18/9. • Cancer types: CRC. • Stages of disease: II & III. • PS: 0-1.	FOLFOX4 85 mg/m^2^ Every 2 weeks Adjuvant	Reduced glutathione* (14)	Placebo (13)	Baseline, at 5th,9th, and 12^th^ cycles or if the patient showed symptoms, and repeated 3 and 6 months after the last cycle
Yehia et al, 2019 (40)	• Country: Egypt. • Ethnicity: not provided. • Mean age: overall: 45.8 ± 9.6, intervention: 45.6 ± 10.5, control: 45.9 ± 8.6. • Sex: M: F: 29/32; intervention: 16/14; control: 13/18. • Cancer types: CRC. • Stages of disease: IV. • PS: not provided.	FOLFOX6 85 mg/m^2^ Every 2 weeks Palliative	L-Carnosine* (30)	Control (31)	3 months
Gobran, 2013 (22)	• Country: Egypt. • Ethnicity: not provided. • Mean age: intervention: 46.5 ± 12, control: 45.9 ± 13.8. • Sex: M: F: 34/26; intervention: 16/14; control: 18/12. • Cancer types: CRC. • Stages of disease: II: intervention (N=11), control: (N=13), III: intervention (N=18), control: (N=17), IV: intervention (N=1), control: (N=0). • PS: 0-1.	FOLFOX4, mFOLFOX6, FLOX 85 mg/m^2^ Every 2 weeks Adjuvant & Palliative	Ca^2+^/Mg^2+^ before and after Chemotherapy (30)	Placebo (30)	Baseline and at each 2-week cycle
Grothey et al, 2011 (23)	• Country: USA. • Ethnicity: predominantly White (96%). • Age: <65: intervention (N=33), control: (N=33). • Sex: M: F: 54/48; intervention: 27/23; control: 27/25. • Cancer types: CRC. • Stages of disease: II & III. • PS: Not provided.	FOLFOX4, mFOLFOX6 +/- bevacizumab or Cetuximab 85 mg/m^2^ Every 2 weeks Adjuvant	Ca^2+^/Mg^2+^ before and after Chemotherapy (50)	Placebo (52)	Baseline and at each 2-week cycle
Ishibashi et al, 2010 (24)	• Country: Japan. • Ethnicity: not provided. • Age (median and range): intervention: 63 (32–74), control: 64 (35–73). • Sex: M: F: 16/17; intervention: 9/8; control: 7/9. • Cancer types: CRC. • Stages of disease: IV. • PS: 0-2.	mFOLFOX6 85 mg/m^2^ Every 2 weeks Adjuvant & Palliative	Ca^2+^/Mg^2+^ before and after Chemotherapy (17)	Placebo (16)	Baseline and at each 2-week cycle
Loprinzi et al, 2014 (25)	• Country: USA. • Ethnicity: White: intervention 1 (N=96), intervention 2 (N=99), control (N=105), Black or African American: intervention 1 (N=16), intervention 2 (N=15), control (N=10), Asian: intervention 1 (N=4), intervention 2 (N=1), control (N=2), American Indian or Alaska Native: intervention 1 (N=0), intervention 2 (N=1), control (N=1). • Median age: overall: 56, intervention 1: 57, intervention 2: 57, control: 56. • Sex: M: F: 169/184, intervention 1 (56/62), intervention 2 (56/60), control: (57/62). • Cancer types: CRC. • Stages of disease: II: intervention 1 (N=22), intervention 2 (N=22), control (N=22), III: intervention 1 (N=89), intervention 2 (N=88), control (N=88), IV: intervention 1 (N=7), intervention 2 (N=6), control (N=9). • PS: Not provided.	FOLFOX4 or mFOLFOX6 85 mg/m^2^ Every 2 weeks Adjuvant	Intervention 1: Ca^2+^/Mg^2+^ before and after chemotherapy (118) Intervention 2: Ca^2+^/Mg^2+^ before and placebo after chemotherapy (116)	Placebo (119)	Baseline and at each 2-week cycle
Kono et al, 2013 (30)	• Country: Japan. • Ethnicity: not provided. • Age (median and range): intervention: 67 (40-88), control: 61 (36-82). • Sex: M: F: 48/41; intervention: 23/21; control: 25/20. • Cancer types: CRC. • Stages of disease: IV: intervention (N=36); control (N=35), non-metastatic: intervention (N=8); control (N=10). • (PS: 0): intervention (N=40); control (N=44), (PS:1): intervention (N=4); control (N=1).	FOLFOX4 or mFOLFOX6 85mg/m^2^ Every 2 weeks Adjuvant & palliative	Goshajinkigan (44)	Placebo (45)	Baseline, every 2 weeks until the 8^th^ cycle, and every 4 weeks thereafter until the 26th week
Oki et al, 2015 (31)	• Country: Japan. • Ethnicity: not provided. • Median age: intervention: 62.4 ± 10.6, control: 60.4 ± 11.5. • Sex: M: F: 99/83 intervention: 48/41; control: 51/42. • Cancer types: CRC. • Stages of disease: III. • (PS: 0): intervention (N=85); control (N=92), (PS:1): intervention (N=4); control (N=1).	mFOLFOX6 85mg/m^2^ Adjuvant	Goshajinkigan (89)	Placebo (93)	Baseline and at each 2-week cycle
Aghili et al, 2023 (32)^≠^	• Country: Iran. • Ethnicity: not provided. • Mean age: intervention: 53 ± 12.4, control: 55 ± 11.6. • Sex: M: F: 24/8; intervention: 12/5; control: 12/3. • Cancer types: CRC: intervention (N=17); control (N=13), Oesophageal cancer: intervention (N=0); control (N=2). • Stages of disease: IV: intervention (N=2); control (N=4), non-metastatic: intervention (N=15); control (N=11). • PS: 0-1.	CAPOX 130 mg/m^2^ Every 3 weeks Adjuvant & palliative	Duloxetine (17)	Placebo (15)	1 day before and within 1 week after each cycle
Kobayashi et al, 2020 (41)	• Country: Japan. • Ethnicity: not provided. • Mean age: intervention: 58.1 ± 2.7, control: 67.5 ± 1.7. • Sex: M: F: 11/17; intervention: 8/6; control: 3/11. • Cancer types: CRC. • Stages of disease: II: intervention (N=0); control (N=3), III: intervention (N=9); control (N=7), IV: intervention (N=5); control (N=4). • PS: 0-1.	mFOLFOX6 85mg/m^2^ Adjuvant & palliative	Cystine and theanine (14)	Control (14)	Baseline and at each 2-week cycle
Kotaka et al, 2020 (33)	• Country: Japan. • Ethnicity: not provided. • Age (median and range): intervention 1: 68.0 (38–78), intervention 2: 66.0 (32–79), control: 68.0 (45–79). • Sex: M: F: 39/40, intervention 1 12/15, intervention 2 11/13, control: 16/12. • Cancer types: CRC. • Stages of disease: II: intervention 1 (N=3), intervention 2 (N=6), control: (N=6), IIIa: intervention 1 (N=17), intervention 2 (N=15), control: (N=16), IIIb: intervention 1 (N=7), intervention 2 (N=3), control: (N=6). • (PS:0): intervention 1 (N=27), intervention 2 (N=24), control (N=25), (PS:1): intervention 1 (N=0), intervention 2 (N=0), control: (N=3).	mFOLFOX6 85mg/m^2^ Every 2 weeks Adjuvant	Intervention1: 1-day ART-123 (27) Intervention2: 3-day ART-123 (24)	Placebo (28)	Every day from day 1 to day 3 of each cycle and on days 15 and 43 of cycle 12
Lee et al, 2023 (39)	• Country: US and Canada. • Ethnicity: White (79.3%). • Mean age: 60.9, Sex: M: F: 1098/1352. • Cancer types: CRC. • Stages of disease: III. • PS: 0: N=1741, 1-2: 709.	FOLFOX 85mg/m^2^ Every 2 weeks Adjuvant	Celecoxib (1228)	Placebo (1222)	Every 3 months for 3 years and subsequently every 6 months for 6 years after random assignment or until disease recurrence.
Liu et al, 2013 (34)	• Country: China. • Ethnicity: not provided. • Mean age: 52.5, ≥50: intervention: 10, control: 7, <50: Intervention: 50, control: 53. • Sex: M: F: 83/37; intervention: 43/17; control: 40/20. • Cancer types: CRC. • Stages of disease: IV. • (PS: 0): intervention (N=34), control: (N=29), (PS:1-2): intervention (N=26), control: (N=31).	FOLFOX4 85mg/m^2^ Every 2 weeks Palliative	Guilongtongluoang (60)	Placebo (60)	Baseline and every two cycles for 6 cycles
Motoo et al, 2020 (42)	• Country: Japan. • Ethnicity: not provided. • Age (median and range): intervention: 62 (35-79), control: 68 (53-79). • Sex: M: F: 31/21; intervention: 16/10; control: 15/11. • Cancer types: CRC. • Stages of disease: III. • (PS: 0): intervention (N=20), control: (N=20), (PS:1): intervention (N=6), control: (N=6).	CAPOX 130 mg/m^2^ Every 3 weeks Adjuvant	Ninjin’yoeito (26)	Control (26)	Baseline and at each 3-week cycle
Wang et al, 2020 (35)	• Country: China. • Ethnicity: not provided. • Median age: intervention: 52, control: 55.5. • Sex: M: F: 111/85; intervention: 53/45; control: 58/40. • Cancer types: CRC. • Stages of disease: II: intervention (N=26); control (N=38), III: intervention (N=72); control (N=60). • PS: Not provided.	mFOLFOX6 85mg/m^2^ Every 2 weeks Adjuvant	Monosialotetraexosyl-ganglioside (96)	Placebo (96)	Baseline and at each 2-week cycle
Zhang et al, 2012 (43)	• Country: China. • Ethnicity: not provided. • Median age: intervention: 55.1, control: 57.3. • Sex: M: F: 52/27; intervention: 25/13; control: 27/14. • Cancer types: CRC. • Stages of disease: II: intervention (N=13); control (N=18), III: intervention (N=25); control (N=23). • PS: Not provided.	CAPOX 130 mg/m^2^ Every 3 weeks Adjuvant	Neurotropin (38)	Control (41)	Every week by an Investigator and every 3 weeks by two clinicians

Δ included gastric cancer patients and CRC patients. M, male; F, female; PS, Performance Status.

*Indicates an agent that has antioxidant scavenger properties or boosts anti-oxidative stress defences.

≠included oesophageal cancer patients as well as CRC patient.

The cancer type, treatment intention and the number of patients in the placebo and intervention groups is indicated for each trial.

The studies were conducted in multiple countries, six in Japan (24, 30, 31, 33, 41, 42), four from China (34, 35, 37, 43), three from Egypt (22, 36, 40), two from each of USA (23, 25), Iran (28, 32) and Italy (29, 38), and one from each of USA and Canada (39) (**Table 1**). A total of 1989 patients were given interventional treatments and 1972 control patients were included. 1917 males versus 2008 females were enrolled into the trials and where ethnicity was reported, the majority were White. In most studies the enrolled patients had stage III or IV CRC. The doses of oxaliplatin used to treat patients were 85 (22–25, 30, 31, 33–41), 100 (29) or 130 (28, 32, 42, 43) mg/m^2^ every 2-3 weeks. 14 different pharmacological interventions were investigated: N-acetylcysteine (28, 37) glutathione (29, 38) metformin (36), L-carnosine (40), Ca^2+^/Mg^2+^ infusions (22–25) goshajinkigan (30, 31) monosialotetrahexosylganglioside (35), cystine and theanine (41), recombinant thrombomodulin (ART-123) (33), guilongtongluofang (34), ninjin’yoeito (42), neurotropin (43), duloxetine (32) and celecoxib (39). Included in two separate meta-analysis were first, N-acetylcysteine, glutathione, metformin and L-carnosine, grouped because each is able to support anti-oxidative stress responses in cells either directly *via* scavenger activity, by inducing antioxidant transcription or by supporting mitochondrial health and function; and secondly, Ca^2+^/Mg^2+^ infusions. OIPN was assessed every 2 weeks in most studies (12/20 studies, **Table 1**; range: weekly to every 3 months).

### Risk of bias

**Figure 2:**
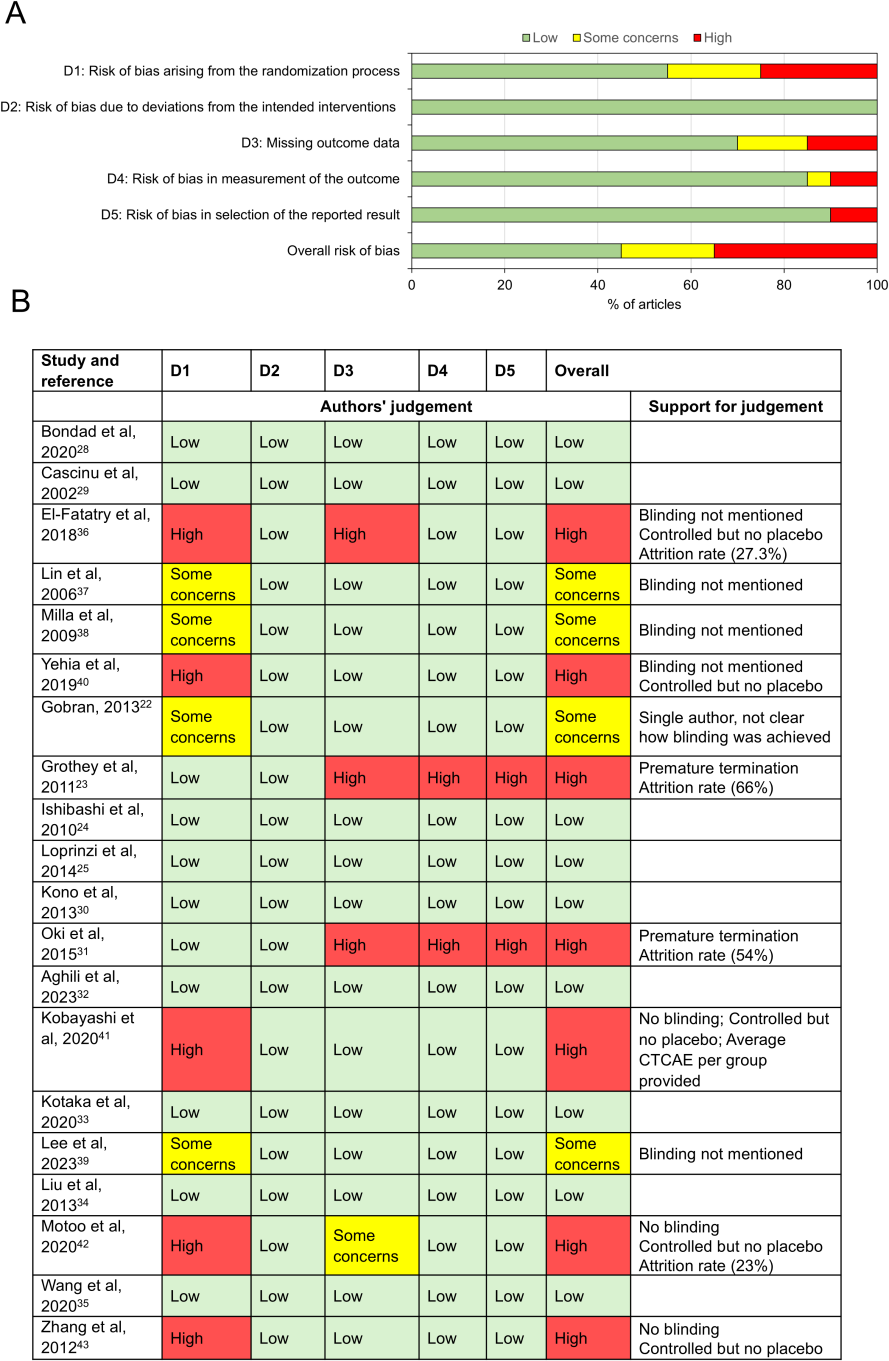
Risk of bias analysis. **(A)** Summary chart to show the proportions of studies with risk of bias in each domain assessed. **(B)** Risk of bias in individual studies with explanations for the authors’ judgement.

The risk of bias analysis showed that 35% of studies had potential high risk of bias with 45% of studies showing low risks of bias (**Figures 2A, B**). Only domain 2, which assessed risk of bias due to deviations from the intended interventions, show that all studies possessed a low risk of bias. However, all other domains showed potential for high risk of bias in 10-35% of the studies.

### Interventions evaluated by single studies

Eight interventions were investigated by only a single study: monosialotetrahexosylganglioside (35), ART-123 (33), duloxetine (32) and celecoxib (39). None showed a significant reduction in grade ≥2 OIPN in clinical trials of 192, 51, 32 and 2,450 patients with CRC, respectively. In the celecoxib trial (39), patients were randomised to 6 or 12 weeks of oxaliplatin ± 3 years of celecoxib treatment. 1,225 patients received celecoxib and there was no difference in incidence of grade ≥2 OIPN in this group compared to the placebo-controlled group either during or after completion of treatment with oxaliplatin. There was also no difference in severity of OIPN measured 12 months after completion of treatment with oxaliplatin using the Functional Assessment of Cancer Therapy/Gynecologic Oncology Group-Neurotoxicity-13 (FACT/GOG-NTX-13). This well-powered trial provides strong evidence against the use of celecoxib to treat OIPN in CRC patients. The other single trials evaluating agents were too small to draw definitive conclusions.

Oral administration of cystine and theanine (41), guilongtongluofang (34), ninjin’yoeito (42) and neurotropin (43) showed statistically significant association with reduced grade ≥2 OIPN (results summarised in **Supplementary Table 3**). The sample sizes employed by these clinical trials ranged from 28-120. Each of these interventions needs further evaluation in future trials.

### Goshajinkigan

Goshajinkigan (TJ-107) is a traditional Japanese herbal remedy used for pain relief. Previous meta-analyses of studies investigating whether goshajinkigan is effective in reducing taxane-induced PN concluded there was no significant effect (44). We identified two fully blinded, placebo-controlled trials that evaluated whether goshajinkigan could ameliorate OIPN (30, 31). The first, Kono et al., 2013 (30) enrolled 93 patients, of which 89 could be included in the analysis. A non-significant reduction in grade ≥2 and grade ≥3 OIPN at cycle 8 was observed in the arm randomised to receive goshajinkigan (39% *vs.* 51% in placebo arm and 7% *vs.* 13%, respectively). The authors also investigated tumour response in 27 patients receiving goshajinkigan and 23 receiving placebo and observed a non-significant improvement in response in the patients receiving goshajinkigan. The second study, Oki et al., 2015 (31) performed an interim analysis of 142 patients and identified a significantly higher incidence of grade ≥2 OIPN in the arm receiving goshajinkigan compared to placebo (50.6% *vs.* 39%), and a significantly shorter time to grade ≥2 OIPN in the goshajinkigan arm (RR=1.908, 95% CI: [1.81-3.083], p=0.007). The interim analysis was performed using assessments of OIPN up until the time when 50% of the planned recruitment had been achieved. All patients had received at least one cycle of oxaliplatin but a breakdown of the number of cycles patients included in the interim analysis had received was not provided. Since it had been determined that goshajinkigan could not significantly outperform placebo even if the study was allowed to continue, it was terminated early. Collectively, these two studies do not support goshajinkigan being used to treat grade ≥2 OIPN.

### Interventions evaluated by three or more studies

Two interventions trialled in three or more studies were analysed by random-effects meta-analysis. First, multiple therapies able to increase anti-oxidative stress responses or defences in cells were evaluated together in a meta-analysis (28, 29, 36–38, 40). Second, trials of Ca^2+^/Mg^2+^ infusions were evaluated together (22, 23, 25).

### Interventions targeting oxidative stress

**Figure 3:**
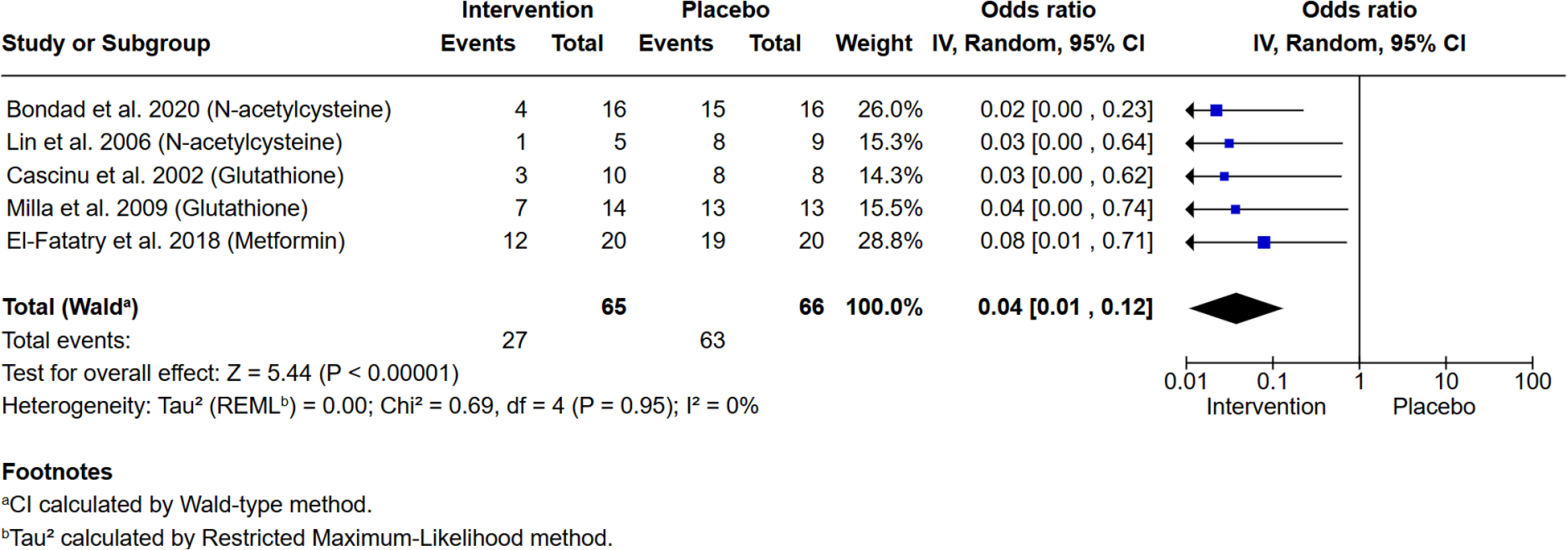
Treatment with anti-oxidative stress drugs is associated with a significant reduction in CTCAE grade ≥2 peripheral neuropathy after 8-12 cycles of oxaliplatin-based chemotherapy. Meta-analysis of 5 studies shows reduced incidence of neuropathy *vs.* placebo (P<0.00001). The intervention tested in each trial is indicated in parentheses.

We analysed 6 trials (28, 29, 36–38, 40) investigating 4 drugs that have anti-oxidative stress effects: N-acetylcysteine (28, 37), glutathione (29, 38), L-carnosine (40) and metformin (36). N-acetylcysteine is a precursor of a key antioxidant, glutathione, and administration of N-acetylcysteine raises cellular glutathione levels. Metformin and L-carnosine induce antioxidant defences indirectly through effects on mitochondria and transcription and L-carnosine also has direct free radical scavenger effects and chelates Zn^2+^ (45, 46). Interestingly each of the studies investigating a drug with anti-oxidative stress effects found significant reduction in CTCAE grade ≥2 OIPN in their intervention arms. Five of the studies assessed OIPN at the end of 8-12 cycles (6 months) therapy (28, 29, 36–38) and three assessed OIPN after 4-6 cycles (2-3 months of treatment) (29, 36, 40) so two separate meta-analyses were performed. In the meta-analysis of the effect on OIPN at 8-12 cycles (6 months of treatment), a statistically significant reduction of large effect was observed (**Figure 3**; OR: 0.04, 95% CI: [0.01-0.12], p<0.00001) with no heterogeneity (I^2^ = 0%). High heterogeneity (I^2^ = 66%) in the meta-analysis of the effect of anti-oxidative stress agents after 4-6 cycles (2-3 months) of treatment with oxaliplatin hindered interpretation, but no significant reduction in grade ≥2 OIPN was observed (**Supplementary Figure 1**; OR: 0.19, 95% CI: [0.02 -1.60], p=0.13).

Given that metformin only has indirect effect on ROS scavenging, we repeated both meta-analyses without the El-Fatatry (36) study. Very similar results were seen after 4-6 cycles (2-3 months) of treatment with oxaliplatin (**Supplementary Figure 2**; OR: 0.10, 95%CI: [0.00-1.98], p=0.13) as well as after 8-12 cycles of treatment (**Supplementary Figure 3**; OR: 0.03, 95%CI: [0.01-0.11], p<0.00001).

Two out of the three studies that tested assessed the effect on OIPN at 4-6 cycles had a control arm rather than a placebo arm and blinding was unclear. A sensitivity analysis assessing the impact of study design was not possible for the 4-6 cycles timepoint. Two of the studies included in the analysis of OIPN at 8-12 cycles were double-blind, placebo-controlled trials: Bondad et al., 2020 (28) and Cascinu et al., 2002 (29), which tested N-acetylcysteine and glutathione respectively. In a meta-analysis including only these two trials and excluding the three trials with unclear blinding, very similar overall measures of effect are seen compared to the 5-study meta-analysis presented in **Figure 3**, (OR: 0.02, 95% CI: [0.00-0.15], p<0.0001; **Supplementary Figure 4**).

### Ca^2+^/Mg^2+^ infusions

**Figure 4:**
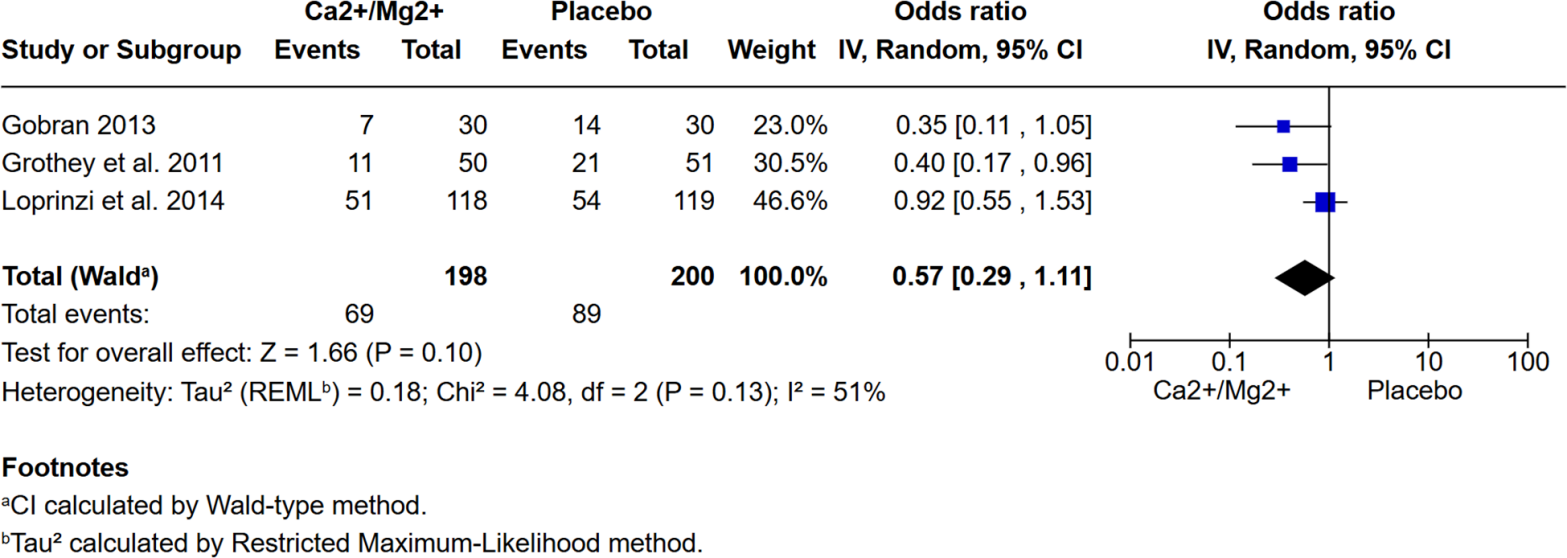
The cumulative incidence of CTCAE grade ≥2 peripheral neuropathy after 6 months of oxaliplatin-based chemotherapy is not reduced by administration of Ca^2+^/Mg^2+^ infusions before and after chemotherapy. Meta-analysis of 3 studies shows no significant difference in the incidence of OIPN *vs.* placebo (P= 0.10).

Three double-blinded, placebo-controlled trials (22, 23, 25) investigated patients randomised to receive either an infusion of Ca^2+^/Mg^2+^ pre- and post- oxaliplatin treatment (198 patients in total) or placebo (200 patients). One rationale for Ca^2+^/Mg^2+^ infusions was that acute PN may be due to the disruption of ion levels that affects neuronal function and homeostasis (47). Meta-analysis of the three trials revealed no association between administration of Ca^2+^/Mg^2+^ infusions pre- and post- oxaliplatin treatment and risk of CTCAE grade ≥2 PN, as assessed after 6 months of oxaliplatin-based chemotherapy (**Figure 4**; OR: 0.57, 95% CI: [0.29- 1.11], p=0.10). This is in keeping with the results of a meta-analysis by Peng et al., 2022 (13). The Loprinzi et al., 2014 (25) trial also included an arm where patients received Ca^2+^/Mg^2+^ infusions before oxaliplatin and placebo after. It also failed to show any significant reduction of OIPN (p=0.3383). The risk of bias analysis (**Figures 2A, B**) identified high risk of bias for the Grothey et al., 2011 study (23) and noted a high attrition rate, as well as premature termination following interim results from the Combined Oxaliplatin Neurotoxicity Prevention Trial (CONcepT) which suggested Ca^2+^/Mg^2+^ infusions affected the response to chemotherapy (48).

## Discussion

In this systematic review we set out to ask whether there is any evidence that existing pharmacological agents or traditional herbal medicines can reduce CTCAE grade ≥2 OIPN in CRC patients. We applied strict inclusion criteria to evaluate the evidence from RCTs that have tested 14 different pharmacological interventions or traditional herbal medicines. Two interventions were subsequently analysed by meta-analysis: first, a group of drugs that all have anti-oxidative stress effects in cells, either directly *via* scavenger activity or indirectly via effects on transcription or on mitochondrial health; and second, Ca^2+^/Mg^2+^ infusions. We found that only anti-oxidative stress drugs were associated with a lower risk of grade ≥2 OIPN when assessed at the end of the treatment (8-12 cycles). There was no significant effect on grade ≥2 OIPN mid-treatment.

Consistent with previous reviews (11–13), no protective effect of Ca^2+^/Mg^2+^ infusions on CTCAE grade ≥2 OIPN was detected. Similarly, there was no evidence of a protective effect of goshajinkigan (TJ-107) in two independent studies (30, 31). All other pharmacological interventions were investigated in only single studies and, with the exception of celecoxib which was investigated in a large trial with a long follow-up (39), all other agents will need further evaluation. Future studies will also need to consider whether treatment with pharmacological agents to reduce OIPN affects the efficacy of the oxaliplatin chemotherapy since most of the RCT reviewed here were too small, or followed up patients for too short a time, to draw conclusions.

A recently published meta-analysis also studied interventions aiming to reduce OIPN (13). **Supplementary Table 4** summarises the details of the 43 studies they included and the reason why each study was, or was not, included in our study. We used stricter inclusion criteria in our study. First, we only analysed data from studies that had assessed OIPN using the CTCAE criteria. Second, we only combined datasets when OIPN was assessed at a similar timepoint in the chemotherapy regime because the severity of PN does change over the course of treatment with oxaliplatin (6). A high proportion of patients (>85%) experience acute neurotoxicity but this often resolves 4-6 weeks into treatment (47). Third, we excluded some studies where we could not access a full text version in English. Fourth, we excluded studies where some patients had baseline PN. Fifth, we excluded studies with fewer than 10 participants available for the final analysis. Twenty studies met our inclusion criteria but the majority of the RCTs recruited small numbers of participants to each arm of their study, with only five studies randomising more than 50 patients to each arm (25, 31, 34, 35, 39). The risk of bias analysis highlights that 7 of the included studies had a high risk of bias in at least one of the assessed categories (23, 31, 36, 40–43).

### The effect of anti-oxidative stress treatments on OIPN

While the beneficial effect of administrating anti-oxidative stress drugs alongside oxaliplatin was clear from the meta-analysis that we performed, the drugs do not all act by the same mechanism.

1. *Glutathione and N-acetylcysteine.* N-acetylcysteine is a precursor of glutathione and administration leads to an increase in glutathione levels in cells (29). Reduced glutathione is a principal scavenger of free radicals (49), hence administration of either N-acetylcysteine or glutathione directly can be considered to act *via* the same mechanism: increasing scavenger levels that reduce reactive species. Interestingly, a study that was excluded from this review because detailed CTCAE counts were not provided, reports a protective effect of administering oral glutamine (15). Glutamine is converted to glutamate –like cysteine, a component of glutathione. While cysteine is usually considered limiting for glutathione production, glutamine levels can drop significantly under stress conditions and may also become limiting. It is possible that the neuroprotective effect seen with glutamine supplementation this trial was also due to an increase in anti-oxidative stress defences.
2. *L-carnosine* (β-alanyl-L-histidine) is a di-peptide synthesised predominantly in muscles and in glia with multiple antioxidant and anti-inflammatory roles (50). L-carnosine acts directly as a scavenger of reactive oxygen and nitrogen species but it also has more indirect effects on cellular defences by inducing Nrf2-dependent transcription. Nrf2 is a key transcription factor that coordinates a large network of anti-oxidative stress transcription *via* the antioxidant response element (ARE) (51). L-carnosine is also able to form a complex with Zn^2+^ to generate polaprezinc which has anti-inflammatory and antioxidant effects, thought in part to be due to upregulation of heme oxygenase (52). Importantly when considering interventions to treat OIPN, L-carnosine is orally bioavailable and brain-penetrant. Having said that, the oral bioavailability of L-carnosine is reduced by the action of serum carnosinases and testing of analogues such as carnosinol that are more resistant to carnosinase activity (53), may be preferable in future trials.
3. *Metformin* (1,1-dimethylbiguanide) is a first-line treatment for type II diabetes and is also commonly prescribed for other glucose-resistance disorders, such as polycystic ovary syndrome (54). It has also been tested in numerous pre-clinical models of acute neurological injury and chronic neurological disease, including as a method of reducing peripheral neuropathy (36). In contrast to the other drugs included in the anti-oxidative stress group, metformin does not have a direct ROS scavenging function. Rather, is has a general effect of increasing cellular oxidative stress defences through several mechanisms and for this reason was included in the meta-analysis. Metformin reduces gluconeogenesis in the liver and increases insulin uptake by cells, although the key biochemical pathways are debated. Metformin can inhibit mitochondrial complex I activity (55), which is likely to be especially relevant to OIPN since oxaliplatin and other platinum drugs have been shown in numerous studies to increase production of reactive oxygen species by mitochondria (8). Inhibition of complex I will also activate AMP-activated protein kinase (AMPK) and subsequently induce autophagy, which has been shown to be neuroprotective in numerous models of neurological disease. Metformin can also activate AMPK more directly *via* Liver kinase B1 (LKB1) (56). Importantly, metformin also alters the redox balance of cells by increasing levels of NADPH; NADPH is required to maintain the cellular pool of reduced glutathione, a key antioxidant. The pleiotropic effects of metformin on cells mean it is not possible to attribute a protective effect against OIPN to any one mode of activity: it is potentially a combination of several. However, if we remove the study that tested metformin (36) from the antioxidant group meta-analysis, we still see a highly significant protective effect at the end of treatment (**Supplementary Figure 3**).

Given that oxaliplatin treatment results in mitochondrial dysfunction and excess production of ROS, it seems probable, therefore, that anti-oxidative stress compounds protect against PN by scavenging excess ROS and/or by inducing Nrf2-based antioxidant transcription. If true, it would be interesting to test targeted forms of antioxidants that concentrate in mitochondria e.g. mitoQ, as these may be more effective at lower doses.

### Treatment of OPIN with anti-oxidative stress drugs

Previous studies have uncovered potentially protective effects of anti-oxidative stress agents against CIPN (57, 58). They included patients with different cancer types, treated with various chemotherapy agents and scored PN using different methods. This study is, to our knowledge, the first to systematically review potentially protective treatments for severe PN in patients with the *same* cancer type, treated with the *same* chemotherapy drug and where the *same* method of scoring PN was used in each trial.

None of N-acetylcysteine, glutathione, L-carnosine or metformin are currently recommended for treating OPIN in the current ASCO guidelines (12). Despite glutathione treatment significantly reducing OIPN in an RCT conducted over 20 years ago (29) none of the anti-oxidative stress agents assessed here have been tested in a large clinical trial. The large effect size of anti-oxidative stress compounds revealed in our meta-analysis, coupled with the lack of heterogeneity in the meta-analysis when OIPN was assessed at 6 months of treatment, supports a larger study to confirm this protective effect. This study must also address potential changes to the efficacy of oxaliplatin. These anti-oxidative stress compounds are cheap and well-tolerated and a successful trial would open options for a simple way to treat OPIN simply with large potential benefit for CRC patients.

### Limitations of the study

The studies included in the anti-oxidative stress therapies meta-analysis were all small and each had a high rate of grade ≥2 OIPN in the placebo arms (88-100%). It is possible that these interventions will not prove effective at reducing incidence of grade ≥2 OIPN if the incidence in the placebo arm is lower. We only considered trials using clinician-scored CTCAE criteria to score OIPN to facilitate comparability across studies. However, we accept that it is a subjective measure and will vary between the scorers and this may not be the optimal measure of OIPN symptom severity. Patient reported outcomes may be preferable, particularly if combined with CTCAE scores (59) and incorporating novel biomarkers such as neurofilament light (NFL) chain has potential for a further objective method for assessing the severity of OIPN (60).

Despite strict inclusion criteria there were potential sources of bias identified in 4 out of the 6 studies that investigated anti-oxidative stress agents (36–38, 40). Blinding was not clearly described for four of the studies (36–38, 40). However, when unblinded trials were excluded, the two remaining studies (28, 29) still showed clear evidence of a significant reduction in grade ≥2 OIPN (*P<*0.0001) in the intervention arm in 50 patients (**Supplementary Figure 4**).

The variation in delivery of the drugs (intravenous and oral) and in trial designs, as well as the high risk of bias in the studies, means that there is an urgent need for further validation through a large-scale, high quality RCT to establish a definitive beneficial effect and to identify which class of antioxidants may be most effective and best tolerated. Despite the promise that antioxidants may help to reduce the severity of OIPN, the evidence for this comes from small trials with differences in trial designs. There remains an urgent need for novel interventions to be developed and tested to reduce peripheral neuropathy and enhance the quality of life for patients receiving oxaliplatin-based therapies.
