# Supplementary material for: Anti-oxidative stress therapies prevent severe chemotherapy-induced peripheral neuropathy in colorectal cancer patients treated with oxaliplatin: a systematic review and meta-analysis
Source: Front Oncol. 2025 Sep 10;15:1642552. doi: 10.3389/fonc.2025.1642552 (PMC12457107; doi:10.3389/fonc.2025.1642552)
Supplement: Supplementary file 1 [file DataSheet1.docx]

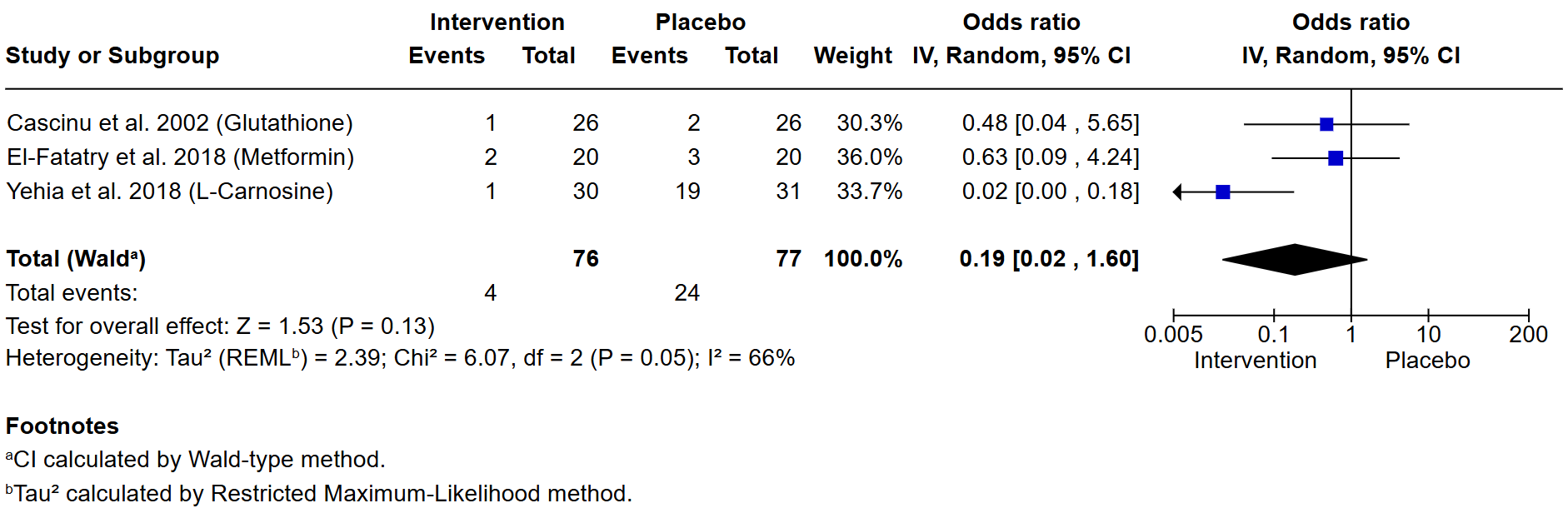


**Supplementary Figure 1. Treatment with anti-oxidative stress drugs is not associated with a reduction in CTCAE grade ≥2 peripheral neuropathy after 4-6 cycles of oxaliplatin-based chemotherapy for colorectal cancer.** Meta-analysis of 3 studies after 2-3 months of chemotherapy shows no significant reduction in incidence of CTCAE grade ≥2 neuropathy *vs.* placebo (P = 0.13). The intervention tested in each trial is indicated in parentheses.


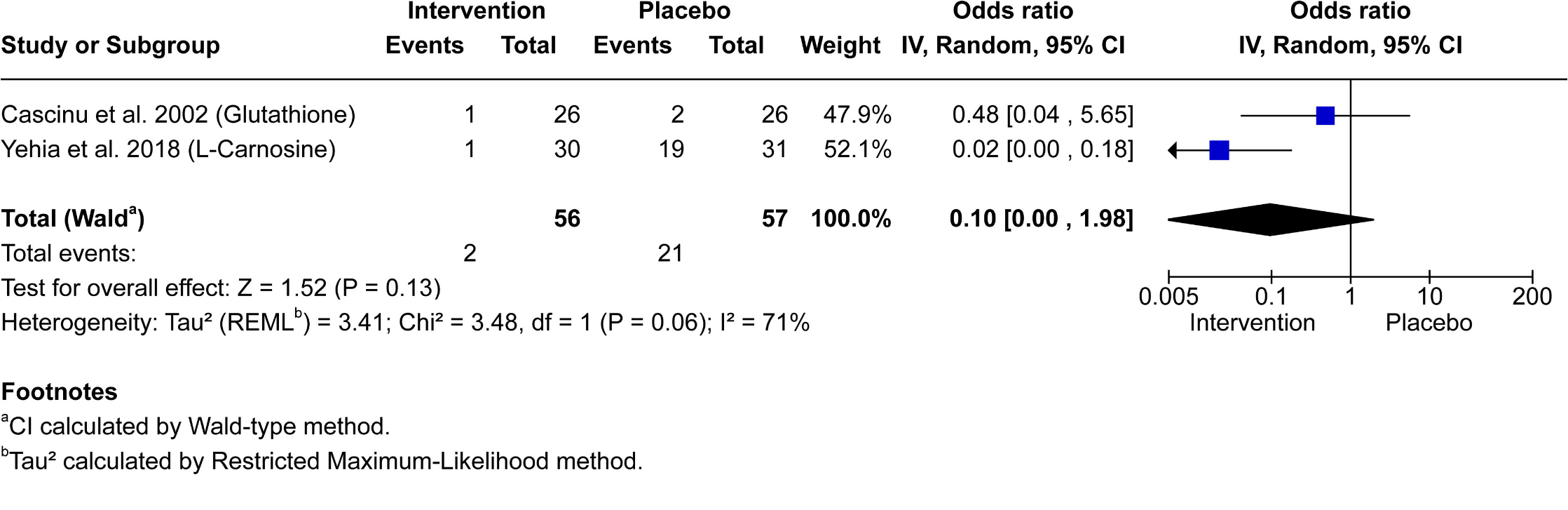


**Supplementary Figure 2. Treatment with anti-oxidative stress drugs with known direct ROS scavenger activity is not associated with a reduction in CTCAE grade ≥2 peripheral neuropathy after 4-6 cycles of oxaliplatin-based chemotherapy for colorectal cancer.** Meta-analysis of 2 studies after 2-3 months of chemotherapy shows no significant reduction in incidence of CTCAE grade ≥2 neuropathy *vs.* placebo (P = 0.13). The intervention tested in each trial is indicated in parentheses.


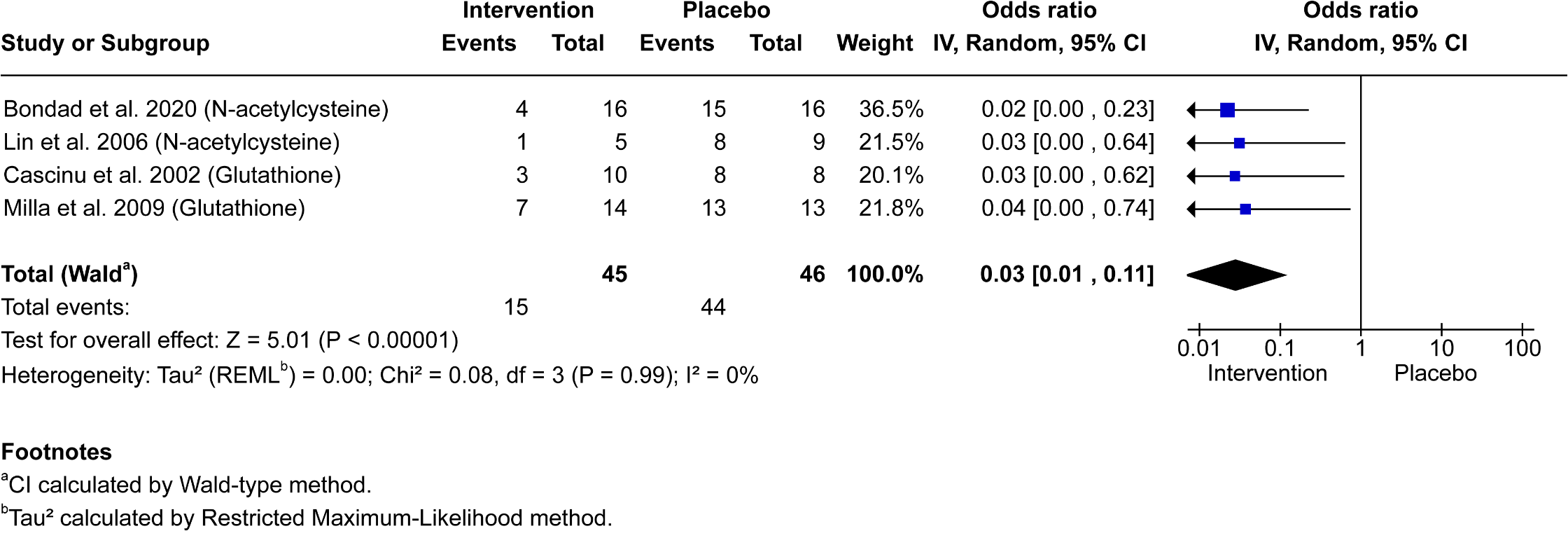


**Supplementary Figure 3. Treatment with anti-oxidative stress drugs with known direct ROS scavenger activity is associated with a significant reduction in CTCAE grade ≥2 peripheral neuropathy after 8-12 cycles of oxaliplatin-based chemotherapy for colorectal cancer.** Meta-analysis of 4 studies that tested the key antioxidant Glutathione or its precursor, N-acetylcysteine, but excluding Metformin shows a highly significant reduction in incidence of CTCAE grade ≥2 peripheral neuropathy at the end of oxaliplatin-based chemotherapy for colorectal cancer (8-12 cycles; P<0.00001). The intervention tested in each trial is indicated in parentheses.


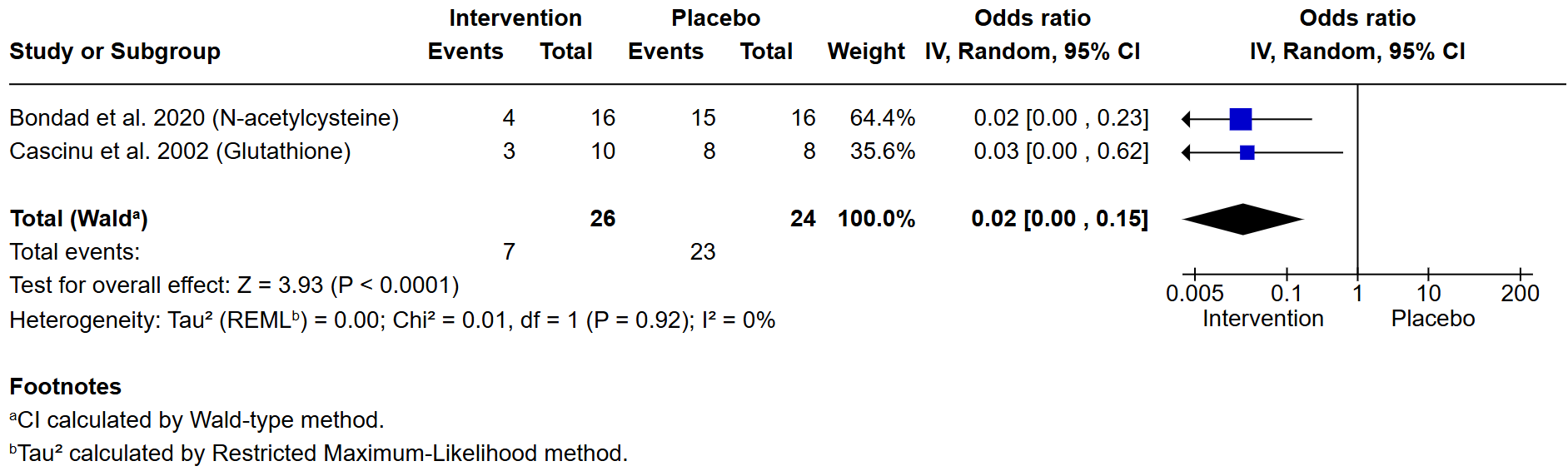


**Supplementary Figure 4. Treatment with anti-oxidative stress drugs N-acetylcysteine and Glutathione, assessed in placebo-controlled double-blinded RCTs is associated with a reduction in CTCAE grade ≥2 peripheral neuropathy after 8-12 cycles of oxaliplatin based chemotherapy for colorectal cancer.** Meta-analysis of 2 studies after 6 months of chemotherapy shows a highly significant reduction in incidence of CTCAE grade ≥2 neuropathy *vs.* placebo (P<0.0001). The intervention tested in each trial is indicated in parentheses.
